# Structures of intermediates during RES complex assembly

**DOI:** 10.1038/srep12545

**Published:** 2015-07-27

**Authors:** Piotr Wysoczanski, Stefan Becker, Markus Zweckstetter

**Affiliations:** 1 Department for NMR-based Structural Biology, Max Planck Institute for Biophysical Chemistry, Am Fassberg 11, 37077 Göttingen, Germany; 2 German Center for Neurodegenerative Diseases (DZNE), 37077 Göttingen, Germany; 3 Center for Nanoscale Microscopy and Molecular Physiology of the Brain, University Medical Center, 37073 Göttingen, Germany

## Abstract

The action of the spliceosome depends on the stepwise cooperative assembly and disassembly of its components. Very strong cooperativity was observed for the RES (Retention and Splicing) hetero-trimeric complex where the affinity from binary to tertiary interactions changes more than 100-fold and affects RNA binding. The RES complex is involved in splicing regulation and retention of not properly spliced pre-mRNA with its three components—Snu17p, Pml1p and Bud13p—giving rise to the two possible intermediate dimeric complexes Pml1p-Snu17p and Bud13p-Snu17p. Here we determined the three-dimensional structure and dynamics of the Pml1p-Snu17p and Bud13p-Snu17p dimers using liquid state NMR. We demonstrate that localized as well as global changes occur along the RES trimer assembly pathway. The stepwise rigidification of the Snu17p structure following the binding of Pml1p and Bud13p provides a basis for the strong cooperative nature of RES complex assembly.

The key step of producing mature and nuclear export ready mRNA particles involves excision of introns in a process termed splicing^1^. Responsible for the catalysis and orchestration of this process is the spliceosome, a multimegadalton assembly of proteins and snRNAs^1^. Unlike ribosomes at the onset of translation, each complete and active spliceosome needs to assemble on its substrate *de novo* during the splicing cycle. Assembly, disassembly and remodelling of the spliceosome is therefore important^1^,^2^. As part of this dynamic process various subcomplexes of changing composition are formed. In line with an efficient remodelling of the spliceosome, the spliceosomal proteins are believed not to act independently. Instead cooperative binding^3^,^4^,^5^, resulting in a cooperative cascade, might drive spliceosome formation and thus its function^2^.

One of the few protein complexes controlling both splicing and export of pre-mRNA is the *re*tention and *s*plicing (RES) complex^6^. A set of introns, specifically associated with pre-mRNA of proteins engaged in nucleotide metabolism, shows splicing controlled by RES^7^,^8^,^9^. The RES complex is composed of the 17.1 kDa small nuclear ribonucleoprotein-associated protein 17 (Snu17p), the pre-mRNA leakage protein 1 (Pml1p) and the 30.5 kDa bud site selection protein 13 (Bud13p)^10^,^11^. RES was shown to interact with U2 snRNA splicing factor 3B (U2 SF3B) proteins and Bud13p showed chemical crosslinks to human sap homolog 155 (Hsh155)^12^,^13^. In addition, we showed that Snu17p can be crosslinked to pre-mRNA between the branch point and 3′ splice site^12^.

Recently, we determined the three-dimensional structure of the core of the RES trimer composed of Snu17p, Pml1p and Bud13p^12^. We further demonstrated that the RES trimer assembles and forms a ternary complex with RNA in a highly cooperative manner^12^. In order to obtain further insight into the molecular basis of the cooperative nature of RES assembly, we here present the three-dimensional structures of two dimeric intermediates along the RES assembly pathway. The structures of the two intermediates provide insight into the atomistic details of the rearrangements that are required in order to accommodate the two intrinsically disordered protein fragments of Bud13p and Pml1p and reveal the nature of the conformational plasticity of RES intermediates.

## Material and methods

Natural abundance and isotopically labelled ^c^Snu17p (residues 25–138 of Snu17p), ^c^Pml1p (residues 22–42 of Pml1p) and ^hc^Bud13p (residues 215–255 of Bud13p) were prepared as described previously^12^. Samples contained either ^13^C,^15^N-labelled peptide with natural abundance ^c^Snu17p or ^13^C,^15^N-labelled ^c^Snu17p with natural abundance peptide. In case of the ^hc^Bud13p–^c^Snu17p dimer, we also prepared a sample where both components of the dimer were ^13^C,^15^N labelled. For measurement of HN residual dipolar couplings (RDCs), samples were aligned by the addition of Pf1 phage (ASLA Biotech).

NMR experiments were carried out at 35 °C on 600, 700, 800 and 900 MHz Bruker spectrometers. Spectrometers were equipped with cryogenically cooled HCN or room temperature HCN probes. In addition to the protein backbones, which were sequence-specifically assigned previously^12^, the sequence-specific backbone resonance assignment of ^hc^Bud13p and ^c^Pml1p in their dimeric complexes with ^c^Snu17p was performed using 3D HNCA, HNCOCA, HNCACB, HNCO^14^ and ^15^N-edited NOESY-HSQC experiments^14^. We also assigned the sidechains in the dimeric complexes with the help of 3D HcCH-TOCSY^15^, 3D HBCBCGCDHD^16^ and 3D ^13^C-edited NOESY-HSQC (both aromatic and aliphatic) experiments^14^. Inter-molecular NOEs were extracted from ^13^C F1-edited/^13^C-^15^N F3-filtered HSQC-NOESY spectra, from the standard Bruker pulse sequence library, which were recorded with a mixing time of 120 ms, recycle delay of 1 s and 32 scans per increment^17^. A total of 256 points in the indirect ^1^H dimension and 32 in the indirect ^13^C dimensions were acquired. FIDs were processed with NMRPipe^18^ or Topspin (Bruker) and the resulting spectra were analysed using ccpnmr Analysis 2.2.1^19^. The BSD-IPAP-HSQC experiment^20^ was used to measure HN RDCs. A total of 91 RDCs were obtained for ^c^Snu17p in the ^c^Pml1p–^c^Snu17p dimer and 75 were used for the purpose of structure validation. In addition, 53 RDCs of residues located in well-defined regions were used for validation of the ^hc^Bud13p–^c^Snu17p dimer structure out of the total number of 87 obtained. For pairwise analysis of RDC sets, all 91 RDCs observed in the ^c^Pml1p–^c^Snu17p dimer were compared against values observed in the RES trimer. Moreover, 79 (65 for the core RRM and 14 in the unfolded C-terminal α-helix) RDCs observed in ^hc^Bud13p–^c^Snu17p dimer were compared against ^c^RES. Finally, 87 RDCs were compared between the ^hc^Bud13p–^c^Snu17p and ^c^Pml1p–^c^Snu17p dimers. The software PALES was used for RDC analysis^21^.

Structures were calculated in CYANA 3.0^22^ and refined using Xplor-NIH 2.3.4^23^. The maximum upper distance limit was set to 6.5 Å and the reference distance to 4.25 Å. Structure calculations were supplemented by dihedral angle restraints that were derived from backbone chemical shifts using TALOS-N^24^. Only high-confidence values (labelled by TALOS-N as „strong“) were used. Eight cycles of structure calculation using CYANA’s noeassig.py protocol were carried out. Intermolecular NOE contacts, which were extracted from filtered/edited NOESY experiments, were treated separately to the automatic CYANA protocols and were manually refined in an iterative manner. H-bonds in α-helices and β-sheets were identified from the initial structural ensemble and confirmed by H-D exchange in combination with NOEs patterns. The structure, which was closest to the mean, was used as representative of an ensemble.

Unless stated otherwise, statistics and structural comparisons were determined using the well-structured parts of the three RES components, that is residues 32–62/74–108 of ^c^Snu17p and residues 223–238 of ^hc^Bud13p in the ^hc^Bud13p–^c^Snu17p dimer, and residues 32–126 of ^c^Snu17p and residues 26–39 of ^c^Pml1p in the ^c^Pml1p–^c^Snu17p dimer. Structural statistics were calculated using Xplor-NIH^23^ and the ICING server^25^. APBS cut-offs were set to ±2 *kT*/eV in order to visualize the electrostatic potential on a solvent accessible surface^26^. Figures were prepared in PyMOL (http://www.pymol.org/) and VMD-xplor^27^.

## Results and Discussion

### Three-dimensional structures of ^c^Pml1p-^c^Snu17p and ^hc^Bud13p-^c^Snu17p complexes

**Figure 1:**
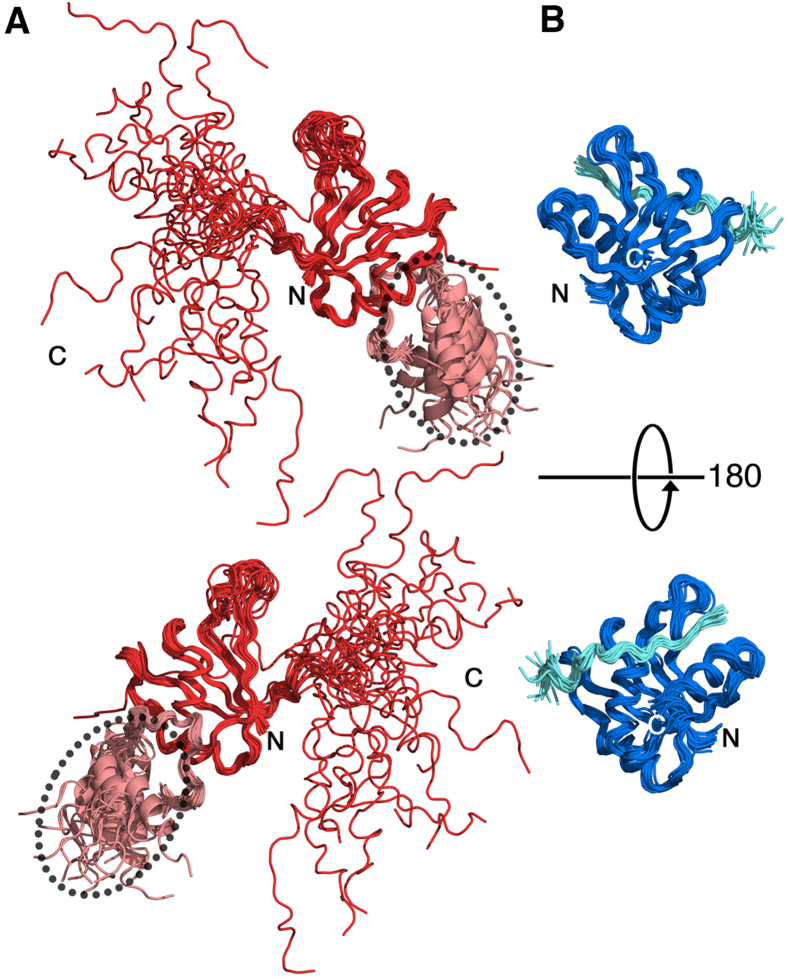
Three-dimensional structures of intermediates during ^c^RES complex assembly. (**A**) ^hc^Bud13p–^c^Snu17p dimer. 20 lowest-energy structures (backbone atoms only) are shown. Residues 116–131 of Snu17p, which fold into an α-helix in the ^c^RES trimer^12^, remain flexible in the ^hc^Bud13p–^c^Snu17p dimer. In addition, the relative orientation of the C-terminal α-helix of ^hc^Bud13p (marked by a dashed ellipsoid) is flexible. Red, ^c^Snu17p; pink ^hc^Bud13p. (**B**) ^c^Pml1p–^c^Snu17p dimer. Shown are the backbones of the 20 lowest-energy structures of the NMR ensemble. Blue, ^c^Snu17p; cyan ^c^Pml1p.

**Figure 2:**
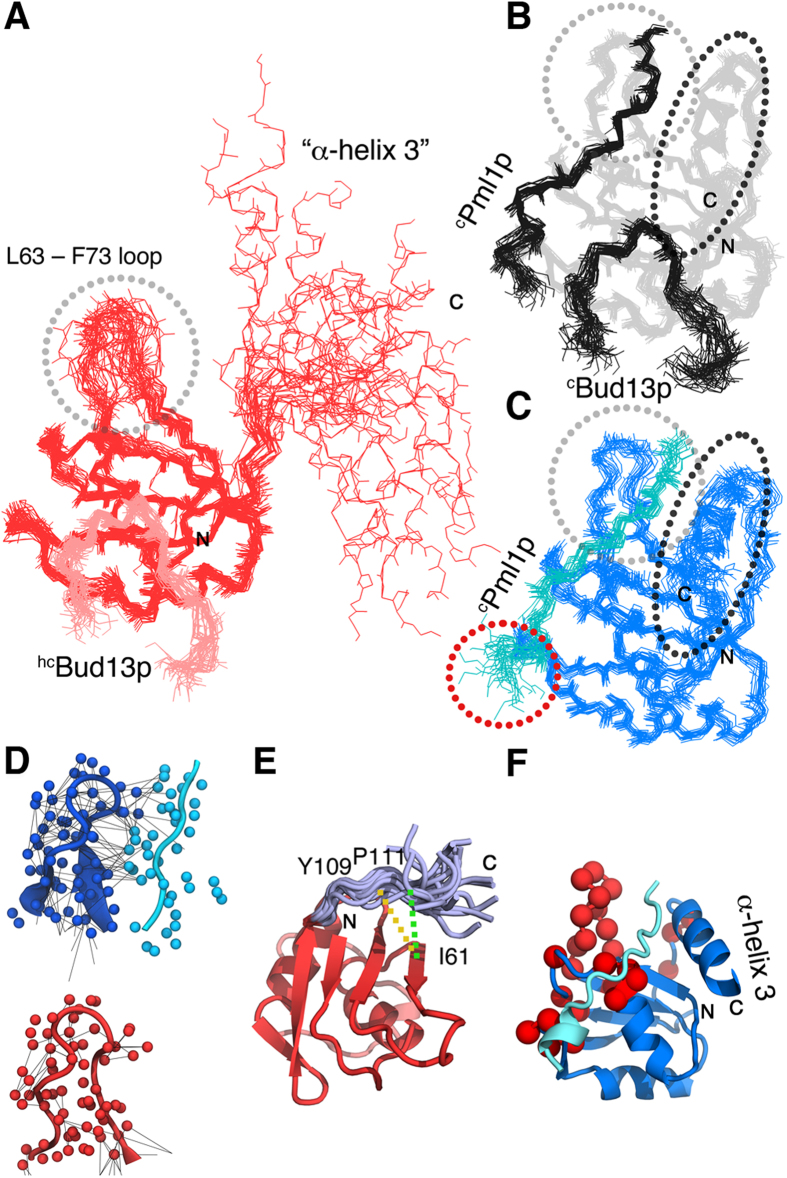
Comparison of the 3D structures of the RES core complex, the ^c^Pml1p–^c^Snu17p dimer and the ^hc^Bud13p–^c^Snu17p dimer. (**A**) ^hc^Bud13p (pink) in complex with –^c^Snu17p (red). (**B**) NMR ensemble of the RES core complex (PDB code: 2MKC^12^). Gray, ^c^Snu17p; black ^c^Bud13p and ^c^Pml1p. (**C**) Structure of ^c^Snu17p (blue) in complex with ^c^Pml1p (cyan). 20 lowest-energy structures (backbone atoms only) are shown. The L63 – F73 loop is encircled in gray and the C-terminal region of ^c^Snu17p, when folded into an α-helix, in black and the disordered C-terminal part of ^c^Pml1p, in red. (**D**) Detailed view of the L63–F73 loop of ^c^Snu17p (blue) and residues 205–210 of ^c^Pml1p (cyan) in the ^c^Pml1p–^c^Snu17p dimer (upper panel). The same loop is shown below for the ^hc^Bud13p–^c^Snu17p dimer. Experimentally observed NOE contacts are represented with black lines. (**E**) Residues 106–115 of Snu17p in the ^hc^Bud13p–^c^Snu17p dimer. Residues 106–115 are shown as an ensemble in light-blue and the rest of the ^hc^Bud13p–^c^Snu17p dimer as a single structure in red. Experimental NOE contacts between Y109 (orange), P111 (green) and I61 are schematically indicated with dashed lines. (**F**) Regions of Snu17p, which are dynamic in the ^hc^Bud13p–^c^Snu17p dimer, were mapped onto the 3D structure of the ^c^Pml1p–^c^Snu17p dimer. Residues 35, 37, 40, 43 – 46, 75, 98, 99, 101, 102, 107, 110, 112 of ^c^Snu17p were marked in red as they showed R_ex_ values exceeding 10 Hz in NMR relaxation measurements^12^ and/or experienced line broadening of 10 Hz above the average value among the folded part of ^c^Snu17p in ^1^H-^15^N HSQC experiments^12^. L63–F73 of ^c^Snu17p was also marked in black to highlight the sparse NOE network as shown in (**D**) and therefore higher RMSD values as presented in (**A**) and consistent with lower than average heteronuclear NOE values reported in^10^. Flexible N- and C-terminal residues (20–32 and 113–138) were excluded from this analysis. α-helix 3 of Snu17p, which is not formed in the ^hc^Bud13p–^c^Snu17p dimer, is labelled and constitutes the most dynamic element in the ^hc^Bud13p–^c^Snu17p dimer structure (as seen in (**A**)).

**Figure 3:**
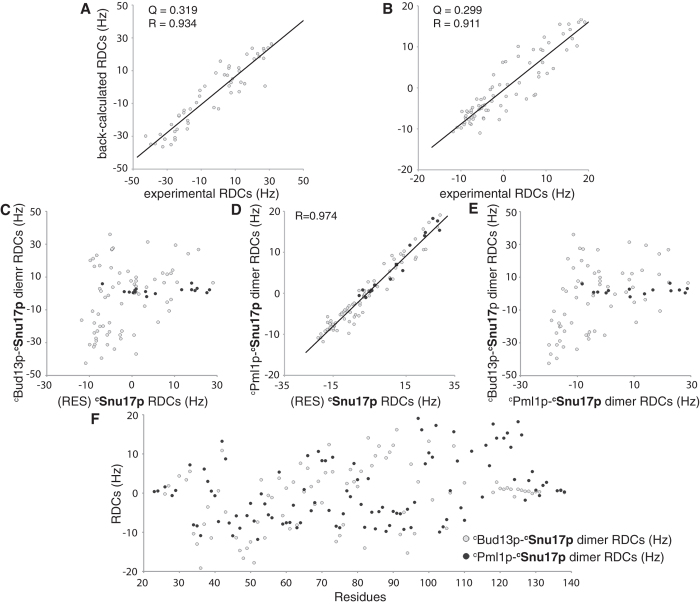
Analysis of HN RDCs observed in ^c^Snu17p when part of the ^c^Pml1p–^c^Snu17p dimer, the ^hc^Bud13p–^c^Snu17p dimer and ^c^RES. (**A,B**) Comparison of experimental RDCs with values back-calculated from the 3D structure of the ^c^Pml1p–^c^Snu17p dimer (**A**) and the ^hc^Bud13p–^c^Snu17p dimer (**B**). (**C,D**) Comparison of RDCs observed in ^c^Snu17p in ^c^RES with RDCs observed in ^c^Snu17p when part of the ^hc^Bud13p–^c^Snu17p dimer (**C**) or the ^c^Pml1p–^c^Snu17p dimer (**D**). Black dots indicate disordered C-terminal residues in the ^hc^Bud13p–^c^Snu17p dimer. (**E**) Comparison of RDCs observed in ^c^Snu17p in complex with ^c^Snu17p and in complex with ^hc^Bud13p. Black dots indicated disordered C-terminal residues in ^hc^Bud13p–^c^Snu17p dimer. (**F**) Residue-specific comparison of RDCs observed in dimeric complexes of ^c^Snu17p with ^c^Pml1p and with ^hc^Bud13p. In (**F**), RDC values of the ^hc^Bud13p–^c^Snu17p dimer were normalized to the magnitude of the alignment tensor of the ^c^Pml1p–^c^Snu17p dimer.

The minimal regions required for binding to residues 25–138 (core Snu17p, ^c^Snu17p) of the 148-residue protein Snu17p are residues 22–42 of Pml1p (^c^Pml1p) and residues 215–245 of Bud13p^12^. The selected regions are sufficient to reproduce the binding affinities of the full-length proteins and thus represent the core of the RES trimer^11^,^12^. In the current study, we further included residues 246–255 of Bud13p (resulting in the fragment ^hc^Bud13p which comprises residues 215–255 of Bud13p) as Collinet *et al*. reported that residues 246–255 form an α-helix^28^. Using a variety of multidimensional NMR experiments the sequence-specific assignment of the ^c^Pml1p–^c^Snu17p dimer as well as the ^hc^Bud13p–^c^Snu17p dimer was achieved. Based on the assignment of 88.0% (^hc^Bud13p–^c^Snu17p dimer) and 91.2% (^c^Pml1p–^c^Snu17p dimer) of all ^1^H proton resonances, we collected a large number of unambiguous intramolecular and intermolecular ^c^Snu17p-^c^Bud13p and ^c^Snu17p-^c^Pml1p NOE distance restraints (Table 1). They defined the structure of the ^c^Pml1p–^c^Snu17p dimer and the ^hc^Bud13p–^c^Snu17p dimer at high resolution (Figs 1 and 2A,C). The final ensembles displayed RMSDs for all heavy atoms of 1.21 Å and 1.31 Å, respectively, with Ramachandran plot statistics of 90.3, 8.5, 1.0, 0.1% (^hc^Bud13p–^c^Snu17p dimer), and 88.2, 11.7, 0.1, 0.0% (^c^Pml1p–^c^Snu17p dimer) for core, allowed, generous and disallowed regions, respectively (Table 1). The quality of the structures was further validated by RDCs (Fig. 3A). Notably, the molecular alignment of Snu17p in Pf1 phage differed strongly between the ^hc^Bud13p-^c^Snu17p dimer and the ^c^RES trimer, as well as between ^hc^Bud13p-^c^Snu17p and ^c^Pml1p-^c^Snu17p dimer, consistent with a release of the C-terminal helix in the ^hc^Bud13p-^c^Snu17p dimer (Supplementary Fig. 1C–E). At the same time, the alignment was nearly identical between ^c^Pml1p-^c^Snu17p dimer and the ^c^RES trimer, in agreement with the stabilization of the C-terminal helix of ^c^Snu17p in the two complexes.

The structure of the ^c^Pml1p-^c^Snu17p dimer and the ^hc^Bud13p-^c^Snu17p dimer retain the β1α1β2β3α2β4 topology of RRMs (Figs 1 and 2) and the domain characteristics of Snu17p seen in the structure of the ^c^RES trimer^12^. Despite the apparent similarity of the Snu17p and Bud13p complex structure to prototypical U2AF homology motif (UHM) and UHM ligand motif ULM interactions, the mode of interaction appears to be different^10^,^12^. Whereas in classical ULM-UHM complexes a central tryptophan is positioned in a deep hydrophobic pocket provided by the RRM domain, tryptophan 232 of Bud13p is found in a shallow space approximately 11 Å away from the canonical site in Snu17p. This is the case for both the ^c^RES trimer as well as ^hc^Bud13p–^c^Snu17p dimer, despite the lack of steric obstruction provided by ^c^Pml1p in the latter case. The charge distribution over all three structures appears to be similar (Supplementary Fig. 1) although, the C-terminal region of Snu17p, which only forms an α-helix in the ^c^Pml1p-^c^Snu17p dimer and the ^c^RES trimer but not in the ^hc^Bud13p-^c^Snu17p dimer (Fig. 1A), is partially positively charged and might contribute to RNA binding. The overall similar charge distribution suggests that optimization of the electrostatic interaction might not be the major contributor to the cooperativity, which was observed for binding of ^c^RES to RNA when compared to monomeric Snu17p and the two dimers^12^.

### Molecular motions in intermediate structures of the RES complex assembly pathway

In a recently solved structure of residues 25–113 of Snu17p in complex with residues 222–256 of Bud13p^10^ the C-terminal region of Snu17p, which forms an α-helix in the ^c^RES trimer and contributes to RNA binding^12^, was not present and therefore did not allow analysis of this functionally important region in the dimeric complex with Bud13p. Based on chemical shift and ^15^N spin relaxation data, we predicted that ^c^Snu17p residues beyond 115 would be unstable in the ^c^Bud13p–^c^Snu17p dimer^12^. Consistent with this prediction, the three-dimensional structure of the ^hc^Bud13p–^c^Snu17p dimer, revealed the C-terminal part of ^c^Snu17p to be disordered and to sample a large conformational space (Figs 1A, 2A). Due to this pronounced mobility, RDC values in this region were efficiently averaged to near zero values (Fig. 3F). However, the long loop, which is formed by residues 106–115 of Snu17p, connects the C-terminal part to the core of Snu17p and traverses its β-sheet in the ^c^RES trimer, remains partially in place in the absence of ^c^Pml1p (Fig. 2E and Supplementary Fig. 2). The partial attachment of this region to the Snu17p β-sheet provides a structural basis for the finding that the ability of the ^c^Bud13p–^c^Snu17p dimer to bind to RNA was diminished but not abolished^12^.

**Figure 4:**
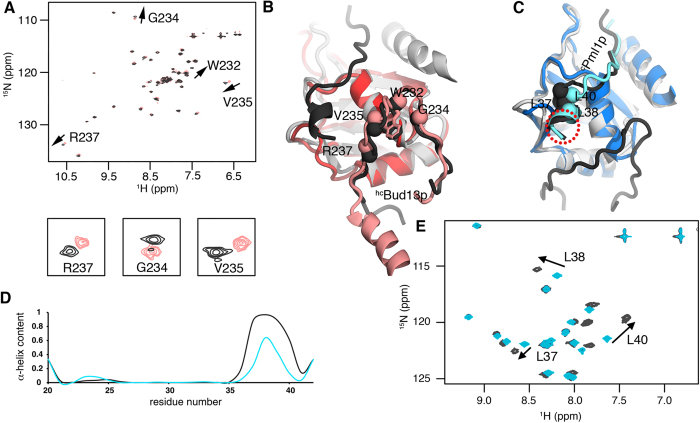
Localized changes in the structure and dynamics during RES core complex assembly. (**A**) Comparison between ^1^H-^15^N HSQC spectra of ^c^Bud13p in ^c^RES (dark gray) and the ^hc^Bud13p–^c^Snu17p dimer (pink). Residues experiencing significant chemical shift perturbation are indicated and some of the corresponding regions are enlarged below. (**B**) Superposition of the 3D structures of the ^c^RES trimer (gray and graphite) and the ^hc^Bud13p–^c^Snu17p dimer (pink and red). Residues experiencing chemical shift perturbation in a) are indicated with spheres. (**C**) Superposition of the 3D structures of the ^c^RES trimer (PDB code: 2MKC^12^; gray and black) and ^c^Pml1p–^c^Snu17p dimer (blue and cyan). The three leucine residues, which strongly shift in (**E**), are highlighted with spheres. The red circle indicates the part of the ^c^Pml1p structure that is partially disordered in the ^c^Pml1p–^c^Snu17p dimer. Structures are colored as follows: graphite, ^c^Bud13p and ^c^Pml1p in ^c^RES; gray, ^c^RES; pink ^c^Bud13p in ^hc^Bud13p–^c^Snu17p dimer; red ^c^Snu17p in ^hc^Bud13p–^c^Snu17p dimer; cyan ^c^Pml1p in ^c^Pml1p–^c^Snu17p dimer; blue ^c^Snu17p in ^c^Pml1p–^c^Snu17p dimer. (**D**) Comparison between the α-helix content of ^c^Pml1p in the ^c^RES trimer (graphite) and the ^c^Pml1p–^c^Snu17p dimer (cyan). The α-helix content was estimated on the basis of backbone chemical shifts using TALOS-N^24^. (**E**) Superposition of ^1^H-^15^N HSQC spectra of ^c^Pml1p in ^c^RES (dark gray) and the ^c^Pml1p-^c^Snu17p dimer (cyan). Three leucine residues experiencing a strong perturbation are marked.

Additional mobility in the ^hc^Bud13p–^c^Snu17p dimer when compared to ^c^RES was observed for the loop between L63 and F73 of ^c^Snu17p, which samples a larger conformational space when ^c^Pml1p is absent (Figs 1A and 2A,C) This can be tracked back to a lack of interactions between residues R64–E66 of ^c^Snu17p and I26, I28 and D31 of ^c^Pml1p as well as sparse intra-loop contacts (Fig. 2D). Altogether, it gives rise to an at least three times lower amount of NOE contacts when ^c^Pml1p is absent (Fig. 2D). Moreover, the L63-F73 loop of ^c^Snu17p was reported to have lower than average heteronuclear NOE values pointing to increased pico-to-nanosecond motions^10^. Although the chemical exchange contribution to the R_2_^15^N relaxation rate (R_ex_) in this region was not elevated, the adjacent loop (V40–E46) showed increased R_ex_ values when compared to the ^c^Pml1p–^c^Snu17p dimer and the ^c^RES trimer^12^. In addition, the β-turn adjacent to V40–E46 showed an elevated R_ex_ contribution and was affected by NMR line broadening^12^. The two loops and the adjacent β-turn are the site of ^c^Pml1p binding, together with the C-terminal region of Snu17p, which folds into an α-helix upon binding of Pml1p (Fig. 2F). The ensemble of Snu17p conformations in this region is therefore ready to accept the incoming Pml1p. On the other hand, we did not detect a structural perturbation of ^hc^Bud13p in the dimeric complex with Snu17p when compared to the ^c^RES trimer. Small ^1^H-^15^N HSQC chemical shift changes (Fig. 4A) were probably caused by a change in the environment associated with the lack of ^c^Pml1p and the unfolding of the C-terminal region of Snu17p (Fig. 4B,C).

Next, we analyzed the differences between the structure of the ^c^Pml1p–^c^Snu17p dimer and the ^c^RES trimer (Fig. 4C). Comparison of the two structures revealed increased disorder of the C-terminal part of ^c^Pml1p associated with a loss of α-helical character (Figs 2B,C and 4C,D). Moreover, the ^c^Pml1p position was slightly modified in response to ^c^Bud13p binding (Fig. 4C). The changes observed in the structure of the ^c^Pml1p–^c^Snu17p dimer were supported by ^1^H-^15^N HSQC spectra: chemical shifts of backbone amides at the C-terminal part of ^c^Pml1p differ depending on the presence of ^c^Bud13p (Fig. 4E) and reflect both changes in the level of disorder and structural changes.

Both ^c^Pml1p and ^hc^Bud13p represent largely the fragments that are necessary for binding to ^c^Snu17p. However, the rest of each sequence could, in the context of the spliceosome, play a role in modulating the assembly of the RES complex. For example, the FHA domain of Pml1p is separated by only a short, six-residue linker from the Pml1p region, which binds to Snu17p. Notably, the phosphopeptide binding site of the FHA domain occurs in proximity to this linker. Alternatively, additional parts of Bud13p, which is intrinsically disordered along its complete sequence, might fold upon binding to other spliceosomal proteins in the context of the spliceosome. Currently, the order of RES complex assembly is not known, but given the two orders of magnitude higher affinity of Bud13p to Snu17p one can speculate that Bud13p might bind first, followed by Pml1p.

### Continuum of UHM-ULM-like interactions

**Figure 5:**
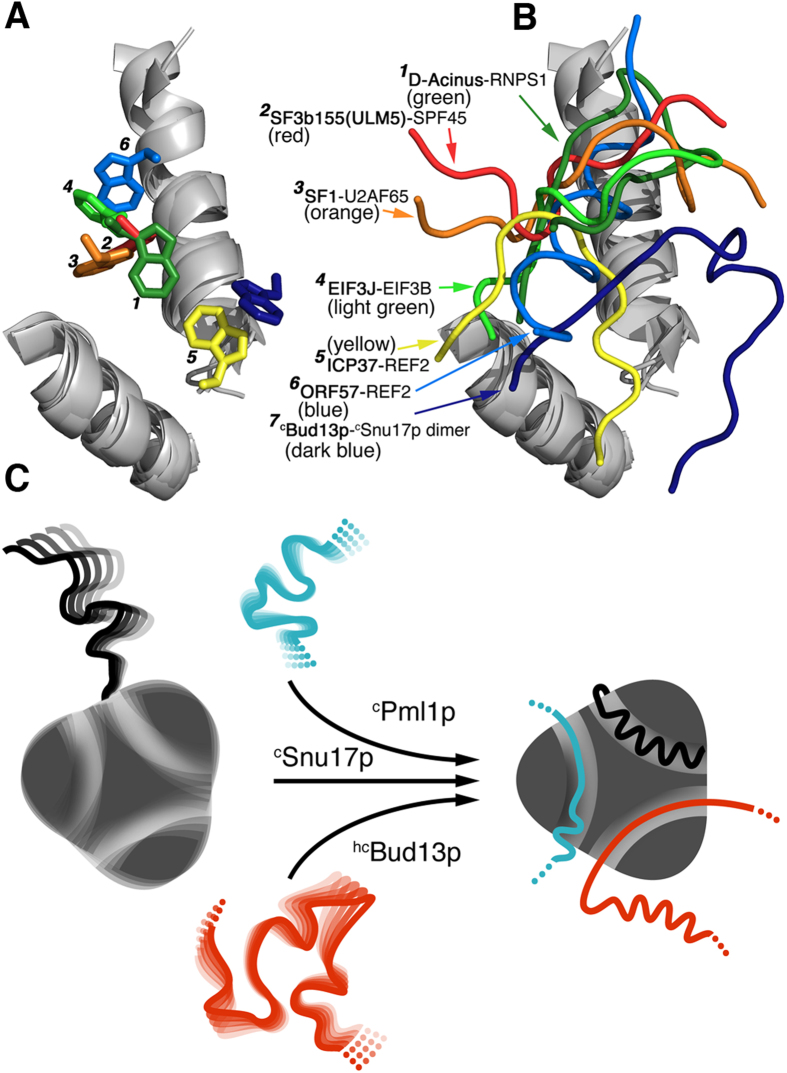
Architecture of central tryptophan containing motifs (CTCMs) in RRMs. (**A**) Comparison between the side chain position of a key tryptophan in different CTCMs bound to RRMs. (**B**) Comparison between backbone traces of CTCMs bound to different RRM domains. CTCMs are labelled 1, dark green, D-Acinus–RNPS1 (PDB code: 4A8X)^31^; 2, red, SF3b155(ULM5)–SPF45 (PDB code: 2PEH)^29^; 3, orange, SF1–U2AF65 (PDB code: 1OPI)^30^; 4, light green, EIF3J–EIF3b (PDB code: 2KRB)^32^; 5, yellow, ICP27–REF2 (PDB code: 2KT5)^33^; 6, light blue, ORF57–REF2 (PDB code: 2YKA)^34^; 7, dark blue, ^hc^Bud13p–^c^Snu17p dimer. Only α-helices 1 and 2 of the RRM are shown for clarity. (**C**) Schematic representation of the folding-upon-binding mechanism as seen in the assembly of the RES complex.

We then compared the non-canonical position of tryptophan 232 of ^c^Bud13p as observed in both the dimeric complex with ^c^Snu17p and the ^c^RES trimer (Fig. 5) and^10^,^12^, with other known RRM-peptide interactions. A canonical UHM–ULM interaction, in which a tryptophan residue is buried in a hydrophobic pocket of the RRM domain was observed for example for the complexes of splicing factor 3b (SF3b155(ULM5)) with alternative splicing factor 45 (SPF45) and of splicing factor 1 (SF1) with the large subunit of U2 snRNP auxiliary factor (U2AF65^29^,^30^). On the other hand, the complex structures of Acinus with RNA binding protein with serine rich domain 1 (RNPS1^31^), eukaryotic translation initiation factors 3 J and 3b (EIF3J–EIF3b^32^), infected cell protein 27 (ICP27) with RNA export factor2 (REF2^33^) and immediate-early phosphoprotein from Saimiriine Herpes Virus ORF57 with REF2^34^ do not have this canonical interaction. In these complexes—as well as in the Snu17p-Bud13p interaction — a conserved tryptophan residue is important for RRM binding, but its position is variable (Fig. 5A). In addition, the part of the protein, which is in contact with the RRM domain, samples a range of conformations (Fig. 5B). Most similar to the ^hc^Bud13p–^c^Snu17p recognition mode is the REF2–ICP27 complex, where the tryptophan side chain occupies a region near the C-terminus of α-helix 2 of the RRM domain (Fig. 5A). Intriguingly, ORF57, another REF2 binder, bares a degree of structural similarity to ^c^Pml1p as observed in the ^c^Pml1p–^c^Snu17p dimer structure. Notably, both Pml1p and REF2 are proteins involved in mRNA export^35^,^36^,^37^. The analysis suggests that there is a structural continuum of how tryptophan containing motifs bind to RRM domains.

In summary, we provided high-resolution structural evidence that the dimeric intermediates along the RES assembly pathway are not a simple structural equivalent of subtracting a given binding partner (Bud13p or Pml1p) of Snu17p from the RES trimeric complex. Instead, a number of localized structural changes are required for successive binding. The local structural changes are further accompanied by the large-scale rearrangement of the C-terminal part of Snu17p, which only folds into a stable α-helix upon interaction with Pml1p. The stepwise rigidification of the Snu17p structure upon binding of Bud13p and Pml1p provides a basis for the strong cooperative nature of RES assembly and RNA binding (Fig. 5C).

## Additional Information

**How to cite this article**: Wysoczanski, P. *et al*. Structures of intermediates during RES complex assembly. *Sci. Rep*. **5**, 12545; doi: 10.1038/srep12545 (2015).

## Supplementary Material

Supplementary Information

## Figures and Tables

**Table 1:** NMR and refinement statistics for the complexes.

	^c^Snu17p (^c^Pml1p)	^c^Pml1p (^c^Snu17p)	^c^Snu17p (^hc^Bud13p)	^hc^Bud13p (^c^Snu17p)
NMR distance and dihedral constraints				
Distance restraints*				
Total NOE	1814	216	1019	322
Intra-residue	467	91	244	109
Inter-residue	1347	125	775	213
Sequential (|*i* – *j*| = 1)	520	96	186	115
Non-sequential (|*i* – *j*| > 1 )	827	29	589	98
Hydrogen bonds	50	—	40	—
Protein–protein intermolecular	228	228	77	77
Total dihedral angle restraints				
Protein				
*ϕ*	100	15	69	27
*ψ*	100	15	69	27
Structure statistics				
Violations (mean and s.d.)				
Distance constraints (Å)	0.021 ± 0.002		0.038 ± 0.004	
Dihedral angle constraints (°)	0.657 ± 0.167		0.766 ± 0.132	
Max. dihedral angle violation (°)	0.7 ± 1.3		0.8 ± 0.8	
Max. distance constraint violation (Å)	0.1 ± 0.4		0.0 ± 0.0	
Deviations from idealized geometry				
Bond lengths (Å)	0.005 ± 0.000		0.004 ± 0.001	
Bond angles (°)	0.540 ± 0.012		0.410 ± 0.019	
Impropers (°)	0.830 ± 0.032		0.314 ± 0.035	
Average pairwise r.m.s. deviation** (Å)				
Protein				
Heavy	1.21 ± 0.11	0.94 ± 0.19	1.14 ± 0.09	1.75 ± 0.15
Backbone	0.69 ± 0.12	0.50 ± 0.14	0.52 ± 0.11	0.65 ± 0.24
Complex				
All complex heavy (C, N, O, P)	1.21 ± 0.10		1.31 ± 0.09	

^*^Excluding intermolecular restraints.

^**^Pairwise r.m.s. deviation was calculated among all refined structures over residues 32–62, 74–108 (^c^Snu17p) and 223–238 (^hc^Bud13p) in the ^hc^Bud13p–^c^Snu17p dimer, and 32–126 (^c^Snu17p) and 26–39 (^c^Pml1p) in the ^c^Pml1p–^c^Snu17p dimer.
