# Supplementary material for: Structures of intermediates during RES complex assembly
Source: Sci Rep. 2015 Jul 27;5:12545. doi: 10.1038/srep12545 (PMC4515762; doi:10.1038/srep12545)
Supplement: Supplementary Information [file srep12545-s1.pdf]

Structures of intermediates during RES complex assembly

Piotr Wysoczanski[1], Stefan Becker[1] and Markus Zweckstetter[1,2,3,*]

[1] Department for NMR-based Structural Biology, Max Planck Institute for Biophysical Chemistry, Am Fassberg 11, 37077 Göttingen, Germany.
[2] German Center for Neurodegenerative Diseases (DZNE), 37077 Göttingen, Germany.
[3] Center for Nanoscale Microscopy and Molecular Physiology of the Brain, University Medical Center, 37073 Göttingen, Germany.

* To whom correspondence should be addressed. Tel: ++49 551 201 2220; Fax: ++49 551 201 2202; Email: Markus.Zweckstetter@dzne.de.

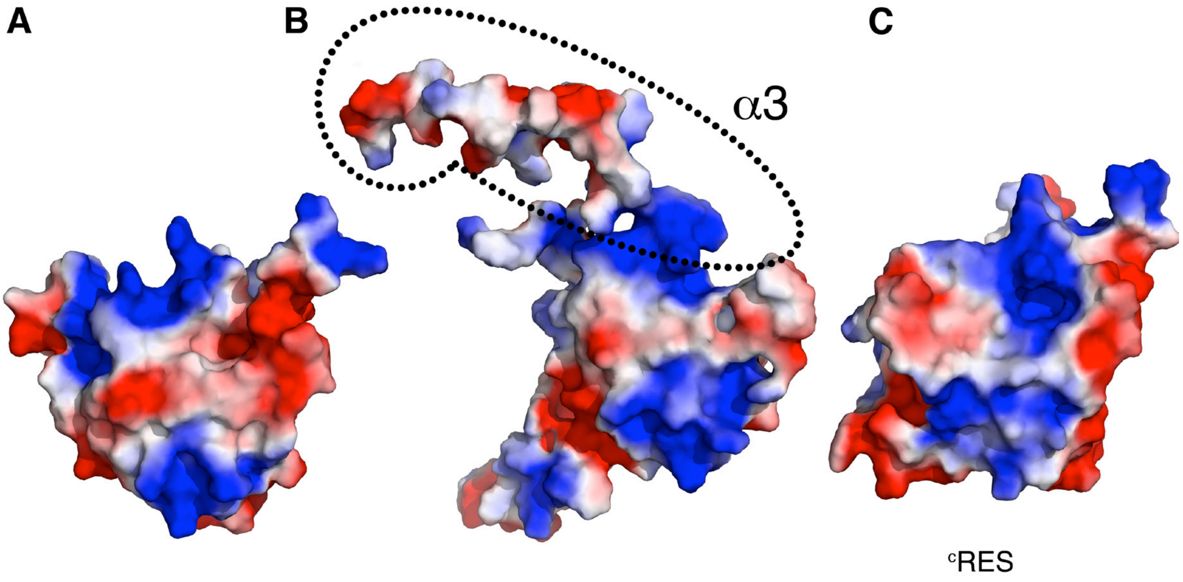

Supplementary Figure 1: Electrostatics of the ^{c}Pml1p–^{c}Snu17p dimer (**A**), the ^{hc}Bud13p–^{c}Snu17p dimer (**B**) and the ^{c}RES trimer (**C**). The C-terminal region of ^{c}Snu17p, which forms an α-helix in the ^{c}Pml1p–^{c}Snu17p dimer and the ^{c}RES trimer, but is largely disordered in the ^{hc}Bud13p–^{c}Snu17p dimer, is encircled.

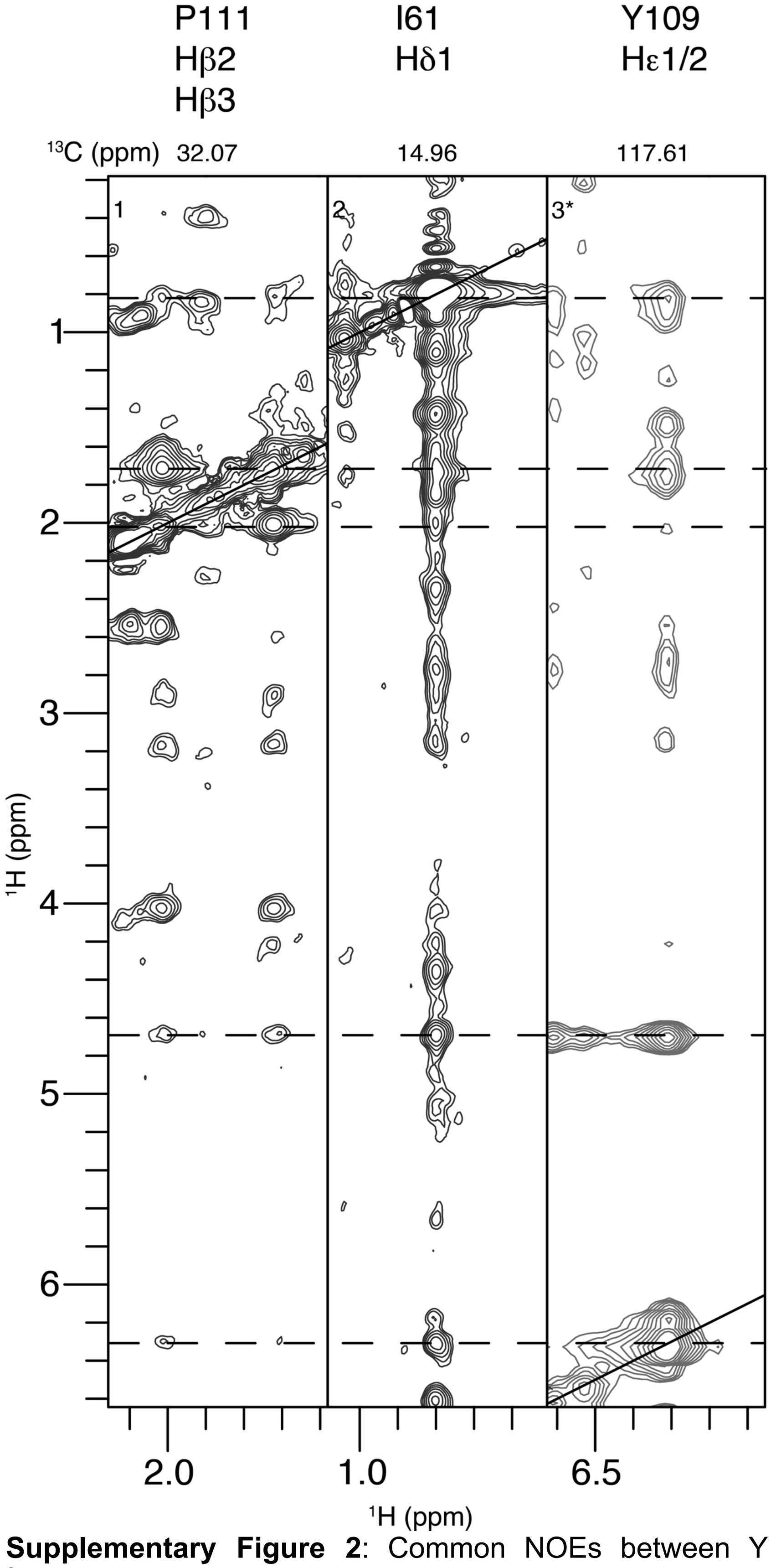

Supplementary Figure 2: Common NOEs between Y109, P111 and I61 of cSnu17p. NOEs are indicated with dashed lines.
